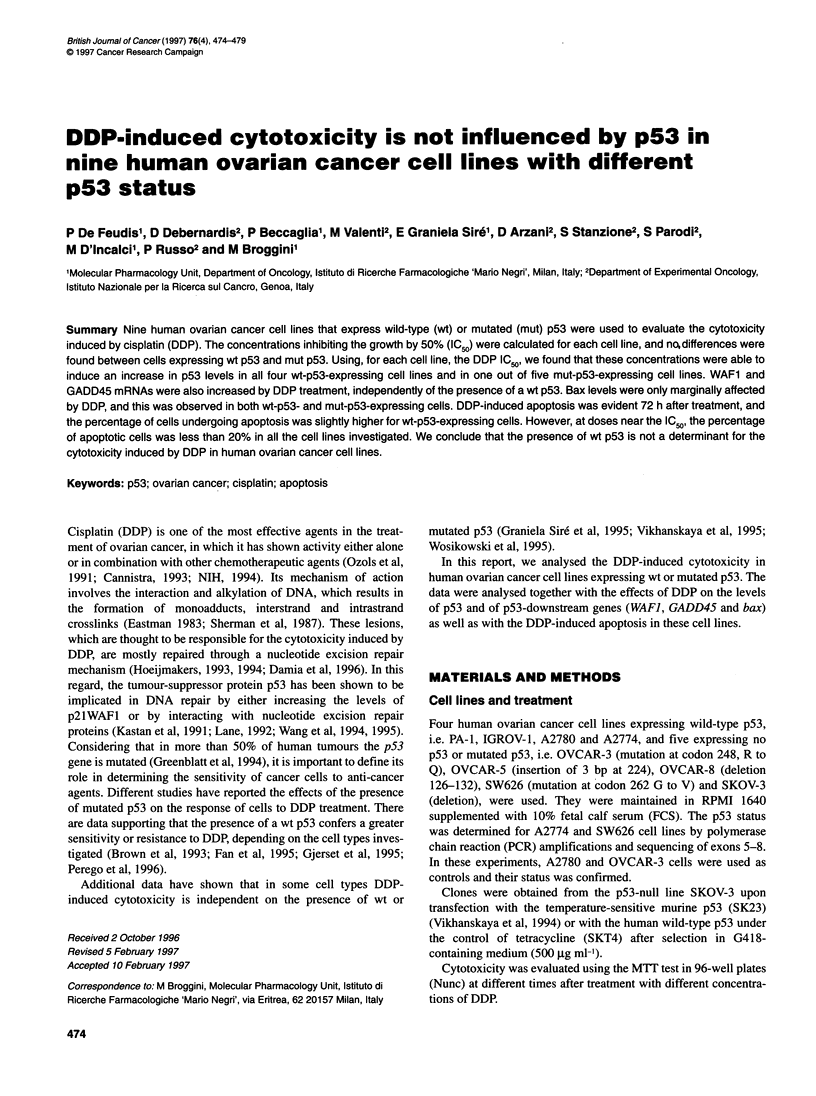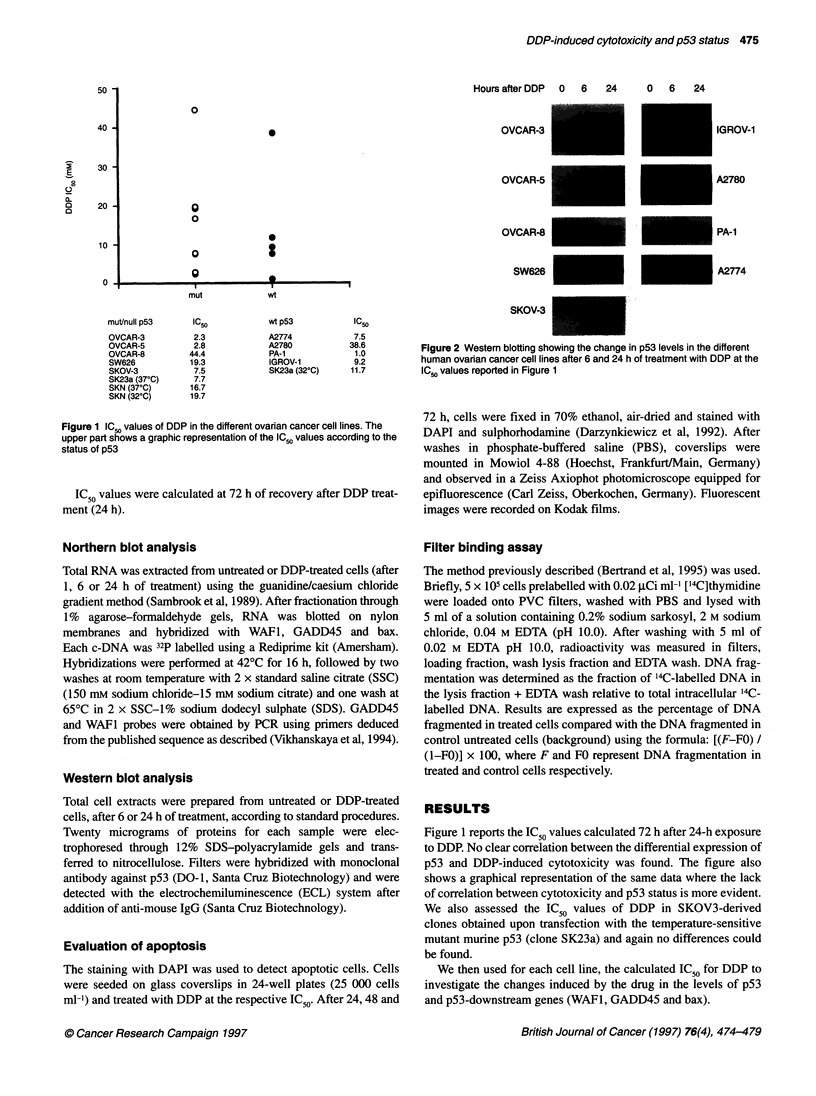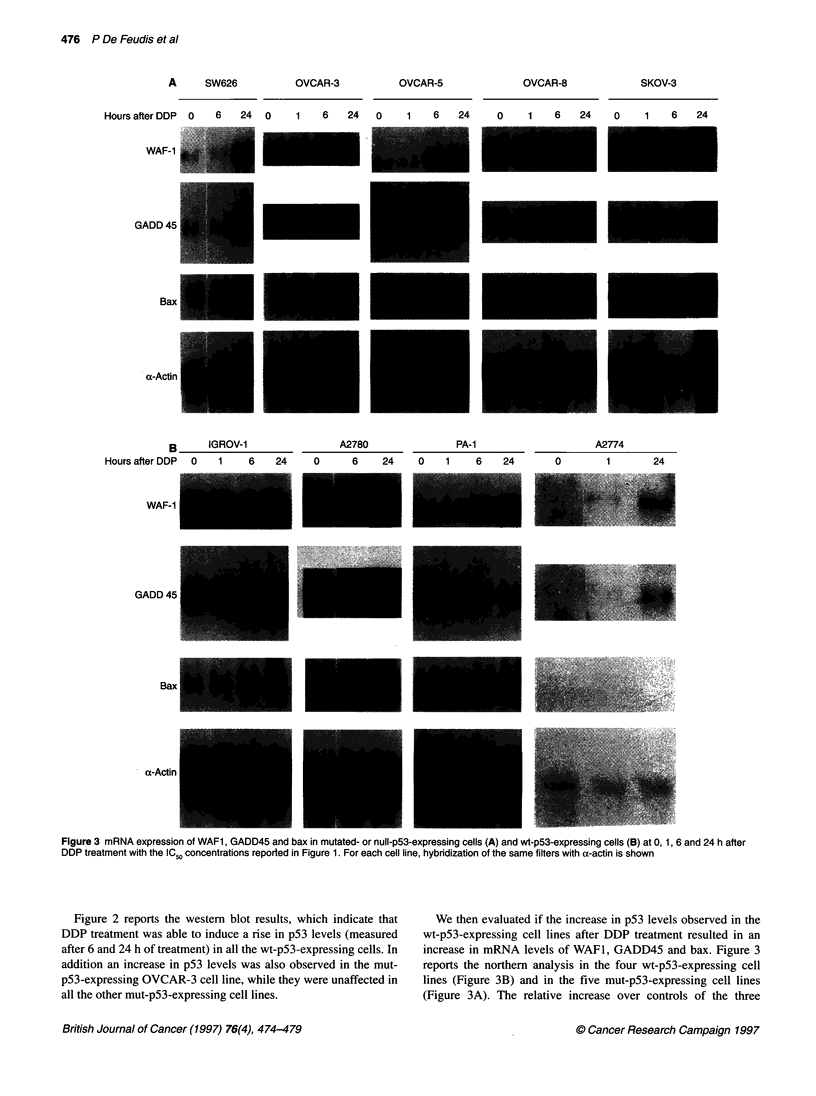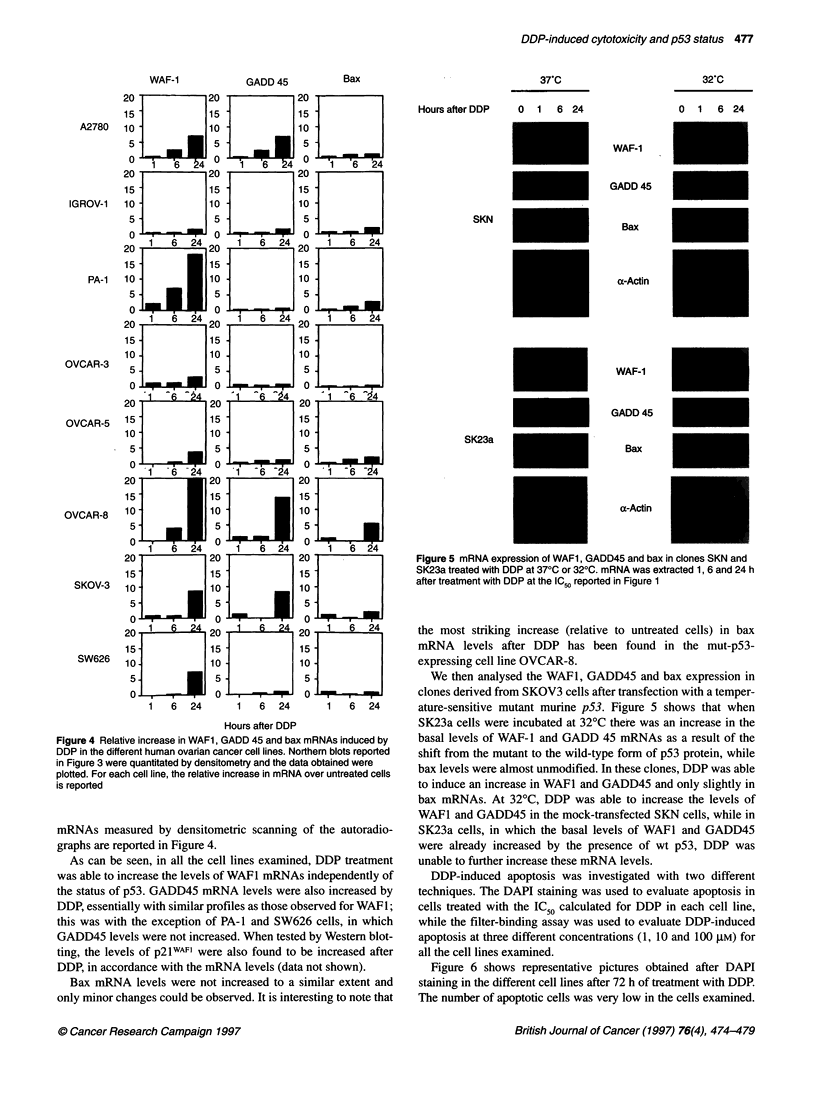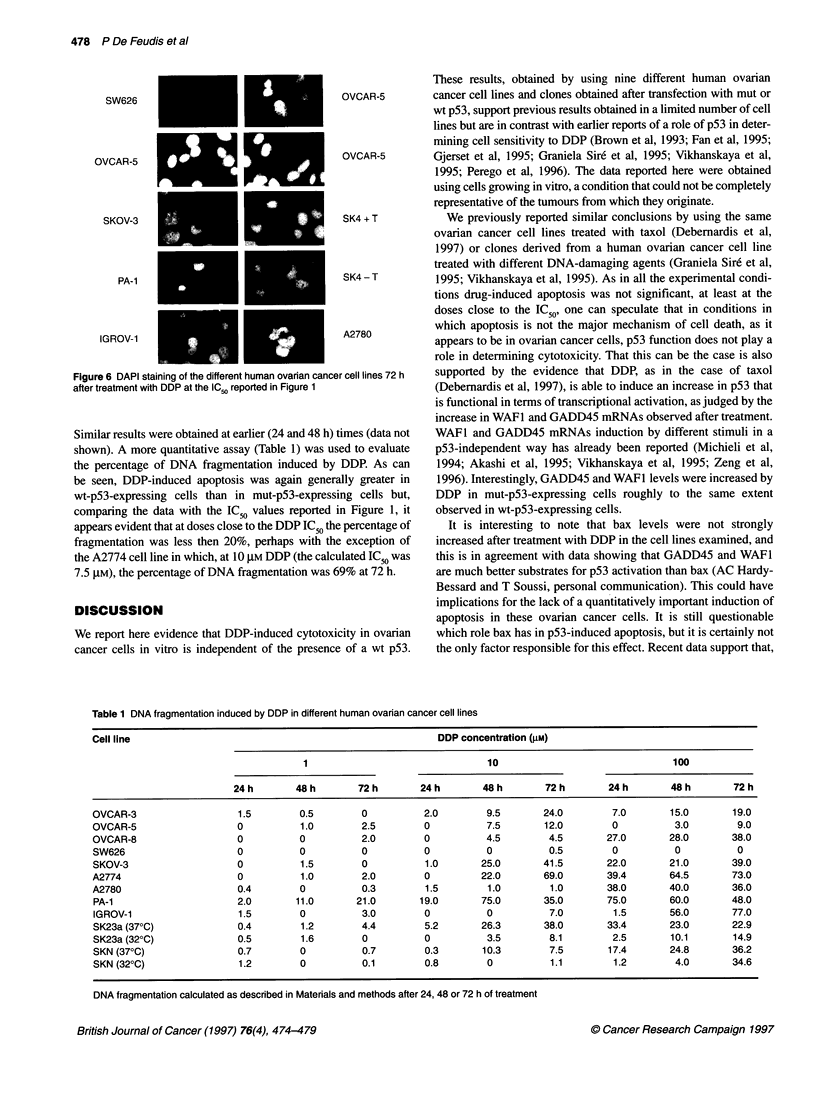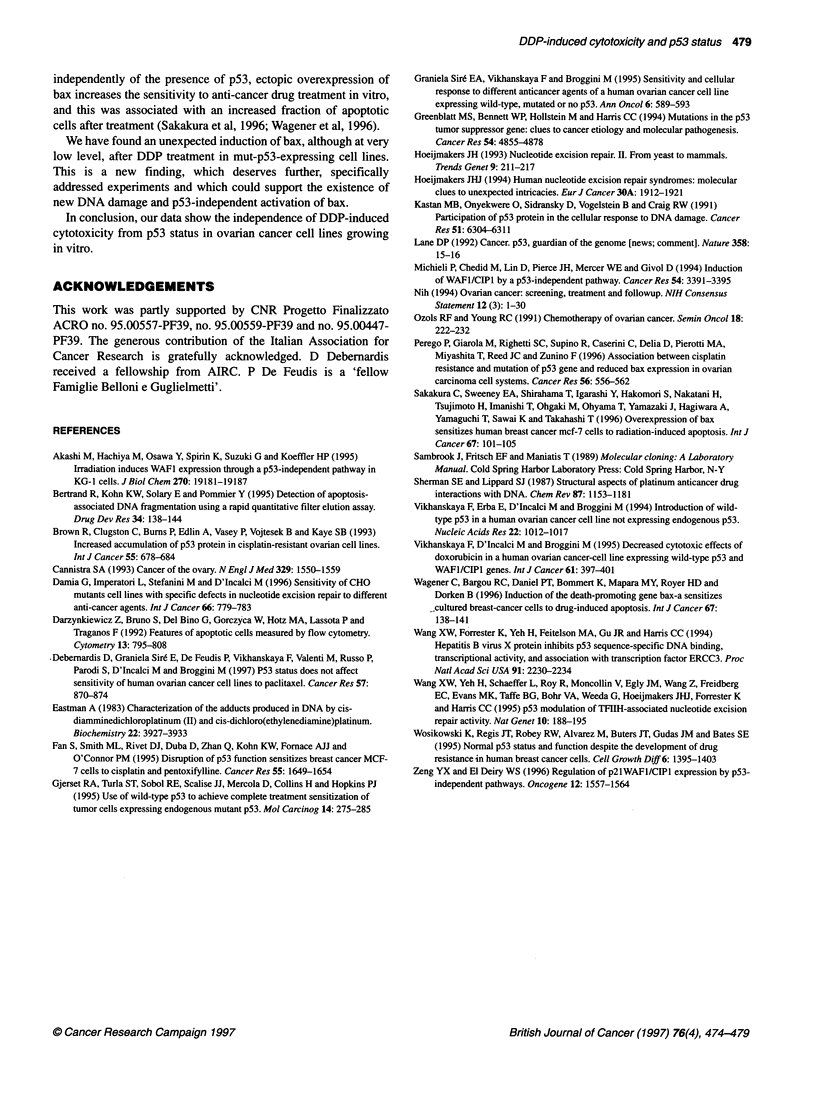# DDP-induced cytotoxicity is not influenced by p53 in nine human ovarian cancer cell lines with different p53 status.

**DOI:** 10.1038/bjc.1997.412

**Published:** 1997

**Authors:** P. De Feudis, D. Debernardis, P. Beccaglia, M. Valenti, E. Graniela SirÃ©, D. Arzani, S. Stanzione, S. Parodi, M. D'Incalci, P. Russo, M. Broggini

**Affiliations:** Department of Oncology, Istituto di Ricerche Farmacologiche Mario Negri, Milan, Italy.

## Abstract

**Images:**


British Journal of Cancer (1997) 76(4), 474–479
© 1997 Cancer Research Campaign

### DDP-induced cytotoxicity is not influenced by p53 in nine human ovarian cancer cell lines with different p53 status

**P De Feudis[1], D Debernardis[2], P Beccaglia[1], M Valenti[2], E Graniela Siré[1], D Arzani[2], S Stanzione[2], S Parodi[2], M D'Incalci[1], P Russo[2] and M Broggini[1]**

[1]Molecular Pharmacology Unit, Department of Oncology, Istituto di Ricerche Farmacologiche 'Mario Negri', Milan, Italy; [2]Department of Experimental Oncology, Istituto Nazionale per la Ricerca sul Cancro, Genoa, Italy

**Summary** Nine human ovarian cancer cell lines that express wild-type (wt) or mutated (mut) p53 were used to evaluate the cytotoxicity induced by cisplatin (DDP). The concentrations inhibiting the growth by 50% ($IC_{50}$) were calculated for each cell line, and no differences were found between cells expressing wt p53 and mut p53. Using, for each cell line, the DDP $IC_{50}$, we found that these concentrations were able to induce an increase in p53 levels in all four wt-p53-expressing cell lines and in one out of five mut-p53-expressing cell lines. WAF1 and GADD45 mRNAs were also increased by DDP treatment, independently of the presence of a wt p53. Bax levels were only marginally affected by DDP, and this was observed in both wt-p53- and mut-p53-expressing cells. DDP-induced apoptosis was evident 72 h after treatment, and the percentage of cells undergoing apoptosis was slightly higher for wt-p53-expressing cells. However, at doses near the $IC_{50}$, the percentage of apoptotic cells was less than 20% in all the cell lines investigated. We conclude that the presence of wt p53 is not a determinant for the cytotoxicity induced by DDP in human ovarian cancer cell lines.

**Keywords:** p53; ovarian cancer; cisplatin; apoptosis

Cisplatin (DDP) is one of the most effective agents in the treatment of ovarian cancer, in which it has shown activity either alone or in combination with other chemotherapeutic agents (Ozols et al, 1991; Cannistra, 1993; NIH, 1994). Its mechanism of action involves the interaction and alkylation of DNA, which results in the formation of monoadducts, interstrand and intrastrand crosslinks (Eastman 1983; Sherman et al, 1987). These lesions, which are thought to be responsible for the cytotoxicity induced by DDP, are mostly repaired through a nucleotide excision repair mechanism (Hoeijmakers, 1993, 1994; Damia et al, 1996). In this regard, the tumour-suppressor protein p53 has been shown to be implicated in DNA repair by either increasing the levels of p21WAF1 or by interacting with nucleotide excision repair proteins (Kastan et al, 1991; Lane, 1992; Wang et al, 1994, 1995). Considering that in more than 50% of human tumours the *p53* gene is mutated (Greenblatt et al, 1994), it is important to define its role in determining the sensitivity of cancer cells to anti-cancer agents. Different studies have reported the effects of the presence of mutated p53 on the response of cells to DDP treatment. There are data supporting that the presence of a wt p53 confers a greater sensitivity or resistance to DDP, depending on the cell types investigated (Brown et al, 1993; Fan et al, 1995; Gjerset et al, 1995; Perego et al, 1996).

Additional data have shown that in some cell types DDP-induced cytotoxicity is independent on the presence of wt or mutated p53 (Graniela Siré et al, 1995; Vikhanskaya et al, 1995; Wosikowski et al, 1995).

*Received 2 October 1996*
*Revised 5 February 1997*
*Accepted 10 February 1997*

*Correspondence to:* M Broggini, Molecular Pharmacology Unit, Istituto di Ricerche Farmacologiche 'Mario Negri', via Eritrea, 62 20157 Milan, Italy

In this report, we analysed the DDP-induced cytotoxicity in human ovarian cancer cell lines expressing wt or mutated p53. The data were analysed together with the effects of DDP on the levels of p53 and of p53-downstream genes (*WAF1*, *GADD45* and *bax*) as well as with the DDP-induced apoptosis in these cell lines.

### MATERIALS AND METHODS

### Cell lines and treatment

Four human ovarian cancer cell lines expressing wild-type p53, i.e. PA-1, IGROV-1, A2780 and A2774, and five expressing no p53 or mutated p53, i.e. OVCAR-3 (mutation at codon 248, R to Q), OVCAR-5 (insertion of 3 bp at 224), OVCAR-8 (deletion 126–132), SW626 (mutation at codon 262 G to V) and SKOV-3 (deletion), were used. They were maintained in RPMI 1640 supplemented with 10% fetal calf serum (FCS). The p53 status was determined for A2774 and SW626 cell lines by polymerase chain reaction (PCR) amplifications and sequencing of exons 5–8. In these experiments, A2780 and OVCAR-3 cells were used as controls and their status was confirmed.

Clones were obtained from the p53-null line SKOV-3 upon transfection with the temperature-sensitive murine p53 (SK23) (Vikhanskaya et al, 1994) or with the human wild-type p53 under the control of tetracycline (SKT4) after selection in G418-containing medium (500 µg $ml^{-1}$).

Cytotoxicity was evaluated using the MTT test in 96-well plates (Nunc) at different times after treatment with different concentrations of DDP.

| mut/null p53 | IC$_{50}$ | wt p53 | IC$_{50}$ |
|---|---|---|---|
| OVCAR-3 | 2.3 | A2774 | 7.5 |
| OVCAR-5 | 2.8 | A2780 | 38.6 |
| OVCAR-8 | 44.4 | PA-1 | 1.0 |
| SW626 | 19.3 | IGROV-1 | 9.2 |
| SKOV-3 | 7.5 | SK23a (32°C) | 11.7 |
| SK23a (37°C) | 7.7 | | |
| SKN (37°C) | 16.7 | | |
| SKN (32°C) | 19.7 | | |

**Figure 1** IC$_{50}$ values of DDP in the different ovarian cancer cell lines. The upper part shows a graphic representation of the IC$_{50}$ values according to the status of p53

**Figure 2** Western blotting showing the change in p53 levels in the different human ovarian cancer cell lines after 6 and 24 h of treatment with DDP at the IC$_{50}$ values reported in Figure 1

IC$_{50}$ values were calculated at 72 h of recovery after DDP treatment (24 h).

### Northern blot analysis

Total RNA was extracted from untreated or DDP-treated cells (after 1, 6 or 24 h of treatment) using the guanidine/caesium chloride gradient method (Sambrook et al, 1989). After fractionation through 1% agarose–formaldehyde gels, RNA was blotted on nylon membranes and hybridized with WAF1, GADD45 and bax. Each c-DNA was $^{32}P$ labelled using a Rediprime kit (Amersham). Hybridizations were performed at 42°C for 16 h, followed by two washes at room temperature with 2 × standard saline citrate (SSC) (150 mM sodium chloride–15 mM sodium citrate) and one wash at 65°C in 2 × SSC–1% sodium dodecyl sulphate (SDS). GADD45 and WAF1 probes were obtained by PCR using primers deduced from the published sequence as described (Vikhanskaya et al, 1994).

### Western blot analysis

Total cell extracts were prepared from untreated or DDP-treated cells, after 6 or 24 h of treatment, according to standard procedures. Twenty micrograms of proteins for each sample were electrophoresed through 12% SDS–polyacrylamide gels and transferred to nitrocellulose. Filters were hybridized with monoclonal antibody against p53 (DO-1, Santa Cruz Biotechnology) and were detected with the electrochemiluminescence (ECL) system after addition of anti-mouse IgG (Santa Cruz Biotechnology).

### Evaluation of apoptosis

The staining with DAPI was used to detect apoptotic cells. Cells were seeded on glass coverslips in 24-well plates (25 000 cells ml$^{-1}$) and treated with DDP at the respective IC$_{50}$. After 24, 48 and 72 h, cells were fixed in 70% ethanol, air-dried and stained with DAPI and sulphorhodamine (Darzynkiewicz et al, 1992). After washes in phosphate-buffered saline (PBS), coverslips were mounted in Mowiol 4-88 (Hoechst, Frankfurt/Main, Germany) and observed in a Zeiss Axiophot photomicroscope equipped for epifluorescence (Carl Zeiss, Oberkochen, Germany). Fluorescent images were recorded on Kodak films.

### Filter binding assay

The method previously described (Bertrand et al, 1995) was used. Briefly, 5 × 10$^{5}$ cells prelabelled with 0.02 μCi ml$^{-1}$ [$^{14}$C]thymidine were loaded onto PVC filters, washed with PBS and lysed with 5 ml of a solution containing 0.2% sodium sarkosyl, 2 M sodium chloride, 0.04 M EDTA (pH 10.0). After washing with 5 ml of 0.02 M EDTA pH 10.0, radioactivity was measured in filters, loading fraction, wash lysis fraction and EDTA wash. DNA fragmentation was determined as the fraction of $^{14}$C-labelled DNA in the lysis fraction + EDTA wash relative to total intracellular $^{14}$C-labelled DNA. Results are expressed as the percentage of DNA fragmented in treated cells compared with the DNA fragmented in control untreated cells (background) using the formula: $[(F\text{–}F0) / (1\text{–}F0)] \times 100$, where $F$ and F0 represent DNA fragmentation in treated and control cells respectively.

### RESULTS

Figure 1 reports the IC$_{50}$ values calculated 72 h after 24-h exposure to DDP. No clear correlation between the differential expression of p53 and DDP-induced cytotoxicity was found. The figure also shows a graphical representation of the same data where the lack of correlation between cytotoxicity and p53 status is more evident. We also assessed the IC$_{50}$ values of DDP in SKOV3-derived clones obtained upon transfection with the temperature-sensitive mutant murine p53 (clone SK23a) and again no differences could be found.

We then used for each cell line, the calculated IC$_{50}$ for DDP to investigate the changes induced by the drug in the levels of p53 and p53-downstream genes (WAF1, GADD45 and bax).

© Cancer Research Campaign 1997

**Figure 3** mRNA expression of WAF1, GADD45 and bax in mutated- or null-p53-expressing cells (**A**) and wt-p53-expressing cells (**B**) at 0, 1, 6 and 24 h after DDP treatment with the $IC_{50}$ concentrations reported in Figure 1. For each cell line, hybridization of the same filters with α-actin is shown

Figure 2 reports the western blot results, which indicate that DDP treatment was able to induce a rise in p53 levels (measured after 6 and 24 h of treatment) in all the wt-p53-expressing cells. In addition an increase in p53 levels was also observed in the mut-p53-expressing OVCAR-3 cell line, while they were unaffected in all the other mut-p53-expressing cell lines.

We then evaluated if the increase in p53 levels observed in the wt-p53-expressing cell lines after DDP treatment resulted in an increase in mRNA levels of WAF1, GADD45 and bax. Figure 3 reports the northern analysis in the four wt-p53-expressing cell lines (Figure 3B) and in the five mut-p53-expressing cell lines (Figure 3A). The relative increase over controls of the three

© Cancer Research Campaign 1997

**Figure 4** Relative increase in WAF1, GADD 45 and bax mRNAs induced by DDP in the different human ovarian cancer cell lines. Northern blots reported in Figure 3 were quantitated by densitometry and the data obtained were plotted. For each cell line, the relative increase in mRNA over untreated cells is reported

**Figure 5** mRNA expression of WAF1, GADD45 and bax in clones SKN and SK23a treated with DDP at 37°C or 32°C. mRNA was extracted 1, 6 and 24 h after treatment with DDP at the $IC_{50}$ reported in Figure 1

mRNAs measured by densitometric scanning of the autoradiographs are reported in Figure 4.

As can be seen, in all the cell lines examined, DDP treatment was able to increase the levels of WAF1 mRNAs independently of the status of p53. GADD45 mRNA levels were also increased by DDP, essentially with similar profiles as those observed for WAF1; this was with the exception of PA-1 and SW626 cells, in which GADD45 levels were not increased. When tested by Western blotting, the levels of $p21^{WAF1}$ were also found to be increased after DDP, in accordance with the mRNA levels (data not shown).

Bax mRNA levels were not increased to a similar extent and only minor changes could be observed. It is interesting to note that the most striking increase (relative to untreated cells) in bax mRNA levels after DDP has been found in the mut-p53-expressing cell line OVCAR-8.

We then analysed the WAF1, GADD45 and bax expression in clones derived from SKOV3 cells after transfection with a temperature-sensitive mutant murine *p53*. Figure 5 shows that when SK23a cells were incubated at 32°C there was an increase in the basal levels of WAF-1 and GADD 45 mRNAs as a result of the shift from the mutant to the wild-type form of p53 protein, while bax levels were almost unmodified. In these clones, DDP was able to induce an increase in WAF1 and GADD45 and only slightly in bax mRNAs. At 32°C, DDP was able to increase the levels of WAF1 and GADD45 in the mock-transfected SKN cells, while in SK23a cells, in which the basal levels of WAF1 and GADD45 were already increased by the presence of wt p53, DDP was unable to further increase these mRNA levels.

DDP-induced apoptosis was investigated with two different techniques. The DAPI staining was used to evaluate apoptosis in cells treated with the $IC_{50}$ calculated for DDP in each cell line, while the filter-binding assay was used to evaluate DDP-induced apoptosis at three different concentrations (1, 10 and 100 μM) for all the cell lines examined.

Figure 6 shows representative pictures obtained after DAPI staining in the different cell lines after 72 h of treatment with DDP. The number of apoptotic cells was very low in the cells examined.

© Cancer Research Campaign 1997

**Figure 6** DAPI staining of the different human ovarian cancer cell lines 72 h after treatment with DDP at the $IC_{50}$ reported in Figure 1

Similar results were obtained at earlier (24 and 48 h) times (data not shown). A more quantitative assay (Table 1) was used to evaluate the percentage of DNA fragmentation induced by DDP. As can be seen, DDP-induced apoptosis was again generally greater in wt-p53-expressing cells than in mut-p53-expressing cells but, comparing the data with the $IC_{50}$ values reported in Figure 1, it appears evident that at doses close to the DDP $IC_{50}$ the percentage of fragmentation was less then 20%, perhaps with the exception of the A2774 cell line in which, at 10 μM DDP (the calculated $IC_{50}$ was 7.5 μM), the percentage of DNA fragmentation was 69% at 72 h.

### DISCUSSION

We report here evidence that DDP-induced cytotoxicity in ovarian cancer cells in vitro is independent of the presence of a wt p53. These results, obtained by using nine different human ovarian cancer cell lines and clones obtained after transfection with mut or wt p53, support previous results obtained in a limited number of cell lines but are in contrast with earlier reports of a role of p53 in determining cell sensitivity to DDP (Brown et al, 1993; Fan et al, 1995; Gjerset et al, 1995; Graniela Siré et al, 1995; Vikhanskaya et al, 1995; Perego et al, 1996). The data reported here were obtained using cells growing in vitro, a condition that could not be completely representative of the tumours from which they originate.

We previously reported similar conclusions by using the same ovarian cancer cell lines treated with taxol (Debernardis et al, 1997) or clones derived from a human ovarian cancer cell line treated with different DNA-damaging agents (Graniela Siré et al, 1995; Vikhanskaya et al, 1995). As in all the experimental conditions drug-induced apoptosis was not significant, at least at the doses close to the $IC_{50}$, one can speculate that in conditions in which apoptosis is not the major mechanism of cell death, as it appears to be in ovarian cancer cells, p53 function does not play a role in determining cytotoxicity. That this can be the case is also supported by the evidence that DDP, as in the case of taxol (Debernardis et al, 1997), is able to induce an increase in p53 that is functional in terms of transcriptional activation, as judged by the increase in WAF1 and GADD45 mRNAs observed after treatment. WAF1 and GADD45 mRNAs induction by different stimuli in a p53-independent way has already been reported (Michieli et al, 1994; Akashi et al, 1995; Vikhanskaya et al, 1995; Zeng et al, 1996). Interestingly, GADD45 and WAF1 levels were increased by DDP in mut-p53-expressing cells roughly to the same extent observed in wt-p53-expressing cells.

It is interesting to note that bax levels were not strongly increased after treatment with DDP in the cell lines examined, and this is in agreement with data showing that GADD45 and WAF1 are much better substrates for p53 activation than bax (AC Hardy-Bessard and T Soussi, personal communication). This could have implications for the lack of a quantitatively important induction of apoptosis in these ovarian cancer cells. It is still questionable which role bax has in p53-induced apoptosis, but it is certainly not the only factor responsible for this effect. Recent data support that,

**Table 1** DNA fragmentation induced by DDP in different human ovarian cancer cell lines

| Cell line | DDP concentration (μM) | | | | | | | | |
|---|---|---|---|---|---|---|---|---|---|
| | 1 | | | 10 | | | 100 | | |
| | 24 h | 48 h | 72 h | 24 h | 48 h | 72 h | 24 h | 48 h | 72 h |
| OVCAR-3 | 1.5 | 0.5 | 0 | 2.0 | 9.5 | 24.0 | 7.0 | 15.0 | 19.0 |
| OVCAR-5 | 0 | 1.0 | 2.5 | 0 | 7.5 | 12.0 | 0 | 3.0 | 9.0 |
| OVCAR-8 | 0 | 0 | 2.0 | 0 | 4.5 | 4.5 | 27.0 | 28.0 | 38.0 |
| SW626 | 0 | 0 | 0 | 0 | 0 | 0.5 | 0 | 0 | 0 |
| SKOV-3 | 0 | 1.5 | 0 | 1.0 | 25.0 | 41.5 | 22.0 | 21.0 | 39.0 |
| A2774 | 0 | 1.0 | 2.0 | 0 | 22.0 | 69.0 | 39.4 | 64.5 | 73.0 |
| A2780 | 0.4 | 0 | 0.3 | 1.5 | 1.0 | 1.0 | 38.0 | 40.0 | 36.0 |
| PA-1 | 2.0 | 11.0 | 21.0 | 19.0 | 75.0 | 35.0 | 75.0 | 60.0 | 48.0 |
| IGROV-1 | 1.5 | 0 | 3.0 | 0 | 0 | 7.0 | 1.5 | 56.0 | 77.0 |
| SK23a (37°C) | 0.4 | 1.2 | 4.4 | 5.2 | 26.3 | 38.0 | 33.4 | 23.0 | 22.9 |
| SK23a (32°C) | 0.5 | 1.6 | 0 | 0 | 3.5 | 8.1 | 2.5 | 10.1 | 14.9 |
| SKN (37°C) | 0.7 | 0 | 0.7 | 0.3 | 10.3 | 7.5 | 17.4 | 24.8 | 36.2 |
| SKN (32°C) | 1.2 | 0 | 0.1 | 0.8 | 0 | 1.1 | 1.2 | 4.0 | 34.6 |

DNA fragmentation calculated as described in Materials and methods after 24, 48 or 72 h of treatment

 © Cancer Research Campaign 1997

independently of the presence of p53, ectopic overexpression of bax increases the sensitivity to anti-cancer drug treatment in vitro, and this was associated with an increased fraction of apoptotic cells after treatment (Sakakura et al, 1996; Wagener et al, 1996).

We have found an unexpected induction of bax, although at very low level, after DDP treatment in mut-p53-expressing cell lines. This is a new finding, which deserves further, specifically addressed experiments and which could support the existence of new DNA damage and p53-independent activation of bax.

In conclusion, our data show the independence of DDP-induced cytotoxicity from p53 status in ovarian cancer cell lines growing in vitro.

### ACKNOWLEDGEMENTS

This work was partly supported by CNR Progetto Finalizzato ACRO no. 95.00557-PF39, no. 95.00559-PF39 and no. 95.00447-PF39. The generous contribution of the Italian Association for Cancer Research is gratefully acknowledged. D Debernardis received a fellowship from AIRC. P De Feudis is a 'fellow Famiglie Belloni e Guglielmetti'.

### REFERENCES

Akashi M, Hachiya M, Osawa Y, Spirin K, Suzuki G and Koeffler HP (1995) Irradiation induces WAF1 expression through a p53-independent pathway in KG-1 cells. *J Biol Chem* **270**: 19181–19187

Bertrand R, Kohn KW, Solary E and Pommier Y (1995) Detection of apoptosis-associated DNA fragmentation using a rapid quantitative filter elution assay. *Drug Dev Res* **34**: 138–144

Brown R, Clugston C, Burns P, Edlin A, Vasey P, Vojtesek B and Kaye SB (1993) Increased accumulation of p53 protein in cisplatin-resistant ovarian cell lines. *Int J Cancer* **55**: 678–684

Cannistra SA (1993) Cancer of the ovary. *N Engl J Med* **329**: 1550–1559

Damia G, Imperatori L, Stefanini M and D'Incalci M (1996) Sensitivity of CHO mutants cell lines with specific defects in nucleotide excision repair to different anti-cancer agents. *Int J Cancer* **66**: 779–783

Darzynkiewicz Z, Bruno S, Del Bino G, Gorczyca W, Hotz MA, Lassota P and Traganos F (1992) Features of apoptotic cells measured by flow cytometry. *Cytometry* **13**: 795–808

Debernardis D, Graniela Siré E, De Feudis P, Vikhanskaya F, Valenti M, Russo P, Parodi S, D'Incalci M and Broggini M (1997) P53 status does not affect sensitivity of human ovarian cancer cell lines to paclitaxel. *Cancer Res* **57**: 870–874

Eastman A (1983) Characterization of the adducts produced in DNA by cis-diamminedichloroplatinum (II) and cis-dichloro(ethylenediamine)platinum. *Biochemistry* **22**: 3927–3933

Fan S, Smith ML, Rivet DJ, Duba D, Zhan Q, Kohn KW, Fornace AJJ and O'Connor PM (1995) Disruption of p53 function sensitizes breast cancer MCF-7 cells to cisplatin and pentoxifylline. *Cancer Res* **55**: 1649–1654

Gjerset RA, Turla ST, Sobol RE, Scalise JJ, Mercola D, Collins H and Hopkins PJ (1995) Use of wild-type p53 to achieve complete treatment sensitization of tumor cells expressing endogenous mutant p53. *Mol Carcinog* **14**: 275–285

Graniela Siré EA, Vikhanskaya F and Broggini M (1995) Sensitivity and cellular response to different anticancer agents of a human ovarian cancer cell line expressing wild-type, mutated or no p53. *Ann Oncol* **6**: 589–593

Greenblatt MS, Bennett WP, Hollstein M and Harris CC (1994) Mutations in the p53 tumor suppressor gene: clues to cancer etiology and molecular pathogenesis. *Cancer Res* **54**: 4855–4878

Hoeijmakers JH (1993) Nucleotide excision repair. II. From yeast to mammals. *Trends Genet* **9**: 211–217

Hoeijmakers JHJ (1994) Human nucleotide excision repair syndromes: molecular clues to unexpected intricacies. *Eur J Cancer* **30A**: 1912–1921

Kastan MB, Onyekwere O, Sidransky D, Vogelstein B and Craig RW (1991) Participation of p53 protein in the cellular response to DNA damage. *Cancer Res* **51**: 6304–6311

Lane DP (1992) Cancer. p53, guardian of the genome [news; comment]. *Nature* **358**: 15–16

Michieli P, Chedid M, Lin D, Pierce JH, Mercer WE and Givol D (1994) Induction of WAF1/CIP1 by a p53-independent pathway. *Cancer Res* **54**: 3391–3395

Nih (1994) Ovarian cancer: screening, treatment and followup. *NIH Consensus Statement* **12** (3): 1–30

Ozols RF and Young RC (1991) Chemotherapy of ovarian cancer. *Semin Oncol* **18**: 222–232

Perego P, Giarola M, Righetti SC, Supino R, Caserini C, Delia D, Pierotti MA, Miyashita T, Reed JC and Zunino F (1996) Association between cisplatin resistance and mutation of p53 gene and reduced bax expression in ovarian carcinoma cell systems. *Cancer Res* **56**: 556–562

Sakakura C, Sweeney EA, Shirahama T, Igarashi Y, Hakomori S, Nakatani H, Tsujimoto H, Imanishi T, Ohgaki M, Ohyama T, Yamazaki J, Hagiwara A, Yamaguchi T, Sawai K and Takahashi T (1996) Overexpression of bax sensitizes human breast cancer mcf-7 cells to radiation-induced apoptosis. *Int J Cancer* **67**: 101–105

Sambrook J, Fritsch EF and Maniatis T (1989) *Molecular cloning: A Laboratory Manual*. Cold Spring Harbor Laboratory Press: Cold Spring Harbor, N-Y

Sherman SE and Lippard SJ (1987) Structural aspects of platinum anticancer drug interactions with DNA. *Chem Rev* **87**: 1153–1181

Vikhanskaya F, Erba E, D'Incalci M and Broggini M (1994) Introduction of wild-type p53 in a human ovarian cancer cell line not expressing endogenous p53. *Nucleic Acids Res* **22**: 1012–1017

Vikhanskaya F, D'Incalci M and Broggini M (1995) Decreased cytotoxic effects of doxorubicin in a human ovarian cancer-cell line expressing wild-type p53 and WAF1/CIP1 genes. *Int J Cancer* **61**: 397–401

Wagener C, Bargou RC, Daniel PT, Bommert K, Mapara MY, Royer HD and Dorken B (1996) Induction of the death-promoting gene bax-a sensitizes cultured breast-cancer cells to drug-induced apoptosis. *Int J Cancer* **67**: 138–141

Wang XW, Forrester K, Yeh H, Feitelson MA, Gu JR and Harris CC (1994) Hepatitis B virus X protein inhibits p53 sequence-specific DNA binding, transcriptional activity, and association with transcription factor ERCC3. *Proc Natl Acad Sci USA* **91**: 2230–2234

Wang XW, Yeh H, Schaeffer L, Roy R, Moncollin V, Egly JM, Wang Z, Freidberg EC, Evans MK, Taffe BG, Bohr VA, Weeda G, Hoeijmakers JHJ, Forrester K and Harris CC (1995) p53 modulation of TFIIH-associated nucleotide excision repair activity. *Nat Genet* **10**: 188–195

Wosikowski K, Regis JT, Robey RW, Alvarez M, Buters JT, Gudas JM and Bates SE (1995) Normal p53 status and function despite the development of drug resistance in human breast cancer cells. *Cell Growth Diff* **6**: 1395–1403

Zeng YX and El Deiry WS (1996) Regulation of p21WAF1/CIP1 expression by p53-independent pathways. *Oncogene* **12**: 1557–1564

© Cancer Research Campaign 1997